# Stop Antibiotics on guidance of Procalcitonin Study (SAPS): a randomised prospective multicenter investigator-initiated trial to analyse whether daily measurements of procalcitonin versus a standard-of-care approach can safely shorten antibiotic duration in intensive care unit patients - calculated sample size: 1816 patients

**DOI:** 10.1186/1471-2334-13-178

**Published:** 2013-04-16

**Authors:** Evelien Assink-de Jong, Dylan W de Lange, Jos A van Oers, Maarten W Nijsten, Jos W Twisk, Albertus Beishuizen

**Affiliations:** 1 Department of Intensive Care, VU University Medical Center, Boelelaan 1117, Amsterdam, 1081 HV, Netherlands; 2 Department of Intensive Care and Emergency Medicine, University Medical Center Utrecht, Heidelberglaan 100, Utrecht, 3584 CX, Netherlands; 3 Department of Intensive Care, St. Elisabeth Hospital Tilburg, Hilvarenbeekseweg 60, Tilburg, 5022 GC, Netherlands; 4 Department of Critical Care, University Medical Center Groningen, Hanzeplein 1, Groningen, 9700 RB, Netherlands; 5 Department of Clinical Epidemiology and Biostatistics, VU University Medical Center, Boelelaan 1117, Amsterdam, 1081 HV, Netherlands; 6 Department of Intensive Care, Medisch Spectrum Twente, Haaksbergerstraat 55, Enschede, 7513 ER, Netherlands

**Keywords:** Antibiotics, Antibiotic duration, Biomarker, Critical illness, C-reactive protein, Defined daily dosage, Intensive care unit, Procalcitonin, Sepsis, Septic shock

## Abstract

**Background:**

Unnecessary long-term use of broad-spectrum antibiotics is linked to the emergence and selection of resistant bacteria, prolonged hospitalisation and increased costs. Several clinical trials indicate that the biomarker procalcitonin (PCT) can guide antibiotic therapy. Some of these trials have shown a promising reduction in the number of antibiotic prescriptions, duration of antibiotic therapy and even length of stay in the ICU, although their size and selection criteria limit their external validity. The objectives of the Stop Antibiotics on guidance of Procalcitonin Study (SAPS) are to evaluate whether daily PCT can improve “real-life” antibiotic use in Dutch ICU’s by reduction of the duration of antibiotic treatment without an increase of recurrent infections and mortality.

**Methods/Design:**

Multicenter randomised controlled intervention trial. Powered for superiority of the primary efficacy endpoint and non-inferiority on the primary safety endpoints (non-inferiority margin is set on 8%).

Inclusion criteria: (1) ICU-patients aged ≥18 years and (2) receiving antibiotics for a presumed or proven infection and (3) signed informed consent. Exclusion criteria: (1) patients who require prolonged antibiotic therapy, (2) suffer from Mycobacterium tuberculosis, (3) cystic fibrosis, (4) viral or parasitic infections and (5) those that are severely immunocompromised or (6) moribund.

The intervention consists solely of an advice to discontinue antibiotic treatment in case PCT has decreased by more than 80% of its peak level (relative stopping threshold) or decrease below a value of 0.5 ng/ml (absolute stopping threshold).

The study hypothesis is that PCT-guided therapy is non-inferior to standard care based on implemented guidelines and local expertise, whilst reducing antibiotic usage. Computerised 1:1 randomisation will allocate 908 patients per arm. Arm 1: standard of care. Arm 2: procalcitonin-guided therapy. The primary efficacy endpoint is consumption of antibiotics expressed as the defined daily dosage and duration of antibiotic therapy expressed in days of therapy. This trial is designed to shorten antibiotics safely, therefore the primary safety endpoint is mortality measured at 28 day and 1 year.

**Discussion:**

This will be the largest procalcitonin-guided antibiotic intervention trial in ICU setting thus far. Currently 1600 of the planned 1816 patients are randomised (November 2012). The first interim analysis has passed without any safety or futility issues.

**Trial registration:**

Trial registration number at http://www.clinicaltrials.gov: Id. Nr. NCT01139489 , at http://www.trialregister.nl: Id.nr. NTR1861.

## Background

Resistance to antimicrobial drugs is an increasing threat to public health. Each year, about 25.000 people die in the EU from an infection with multidrug-resistant bacteria. In the EU infections due to such bacteria result in extra healthcare costs of over 1.5 billion Euros each year [1]. The reasons for the increase in antimicrobial resistance are complex, but it has become evident that unnecessary prolonged use of broad-spectrum antibiotics is linked to the emergence and selection of resistant bacteria, and longer hospitalization [1]. The Netherlands have long been known for its prudent use of antibiotics and its subsequent low number of multi-resistant organisms. However, in the Netherlands in recent years a gradual increase in multi-resistant organisms has been found, mainly located in nursing homes and intensive care units (ICU’s).

In general, the use of different classes of antibiotics has grown steadily in hospitals [2]. In 2000, a survey in Dutch hospitals showed that the overall use of systemic antibiotics was 43 Defined Daily Dose (DDD)/100 patient-days. In 2009 the use of systemic antibiotics had already increased to 70.9 DDD/100 patient-days [3]. In the ICU’s of these hospitals the average use was twice as high (132 DDD/100 patient-days in 2006) [4].

Delayed antibiotic therapy is associated with a worse outcome for patients presenting with a severe acute infection. Each hour of delay in the administration of antibiotic therapy in the first six hours is associated with an average decrease in survival of more than 7% [5]. It is therefore advised to start antibiotics as soon as possible whenever sepsis is considered. Although rapid and adequate antibiotic therapy is of great importance, it is obvious that aggressive strategies can easily lead to a high consumption of broad-spectrum antimicrobial agents. Unfortunately, no objective measures exists on how long antibiotic therapy should be applied. To address the potential overuse of antimicrobial agents, trials have used biomarkers to provide additional objective information about the extent of systemic inflammation and to optimise the duration of antibiotic therapy. Conventional clinical signs such as fever, leukocytosis or increased C-reactive protein (CRP), have proven to be neither specific nor sensitive enough for the diagnosis or treatment of infections in ICU patients.

Over the past two decades procalcitonin (PCT) has been extensively studied as a serum marker of systemic infection and sepsis. Being the precursor of the active hormone calcitonin, PCT is a 116 amino-acid peptide that can be elevated by several orders of magnitude in systemic inflammation accompanying sepsis [6,7]. It has been shown that after trauma or surgery the levels of PCT usually stay below 1 ng/ml [8]. On the other hand, PCT levels that are clearly above 1 ng/ml are often associated with severe bacterial infections that manifest themselves as sepsis and septic shock [6-10].

Previous studies have shown that PCT-guided therapy is not only beneficial for respiratory tract infections [11-14], but also provide useful guidance for antimicrobial treatment in critically ill patients in the ICU who are treated for suspected bacterial infection [15-19]. The large French multicenter PRORATA study demonstrated that patients in the PCT group had significantly more antibiotic free days than patients in the control (usual care) group (14.3 days [SD 9.1] vs. 11.6 days [SD 8.2])[17]. Mortality in the PCT group was non-inferior to that in the control group. Criticism focused on potential treatment bias, unclear adherence to guidelines in the control group, the sample size (630 randomized patients may be underpowered), and especially the 10% non-inferiority margin. The 10% inferiority margin ought to have been a relative measure. However, the PRORATA-study was powered upon a 10% absolute difference in mortality. Such a difference is generally considered unacceptable. Therefore, safety issues remain and it is also not clear whether such a PCT-guided strategy is effective in a setting with relatively low antibiotic consumption and low resistance such as the Netherlands. PCT-guided antibiotic therapy might be beneficial to patients but also to the society at large, because it may lead to lower costs, fewer side effects originating from antibiotic usage and, in time, potentially a decrease in multidrug-resistance. Thus, we designed a large multicenter Dutch PCT-guidance trial to address all of these issues, in particular the optimisation of antibiotic therapy duration in critically ill patients on the ICU, currently solely based on empirical guidelines.

## Methods and design

### Study aim

The objective of this trial is to establish whether the PCT-guided strategy is superior to standard of care based on existing experience, expertise and implemented guidelines. The primary efficacy endpoint is that PCT-guided strategy is superior in terms of antibiotic use, expressed as the Defined Daily Dosage (DDD), and duration of antibiotic therapy expressed in days of therapy. As this trial is designed to shorten antibiotics safely, the primary safety endpoint will be overall mortality measured at 28 days and at 1 year.

Secondary endpoints are reinfection rate, length of stay in the ICU and cost effectiveness.

### Study design and setting

This is a nationwide multicenter prospective, randomised, controlled, open intervention trial performed in 16 Dutch surgical and medical ICU’s.

The Ethics Committee of the VU University Medical Center Amsterdam, Netherlands, approved the study protocol which is in full compliance with the Helsinki declaration. The study is coordinated by a steering committee (the authors), consisting of intensivists from participating ICU’s, who all contributed to the design and execution of this trial. Furthermore, a statistician, an epidemiologist and a pharmacist support the steering committee.

The trial is supervised by an independent safety monitoring board that is not involved in the design and conduct of the trial, or in the recruitment of patients. The board consists of a pulmonologist, an intensivist and a statistician.

### Stratification, randomisation and blinding

Patients will be stratified by diagnosis and centre. Stratification will be performed according to the diagnosis of sepsis, severe sepsis and septic shock. Allocation of patients to either treatment group is concealed by using a centralised randomisation procedure with a computer generated list produced by an independent research organisation, the Julius Center for Human Research, Utrecht, the Netherlands.

### Patients

Included will be patients with an age >18 years, and for whom antibiotics were initiated for an assumed or proven infection on admission or during ICU admission. Current guidelines, like the Surviving Sepsis guidelines, advocate swift initiation of antibiotics whenever infection/sepsis is considered [5]. This study will not interfere with this decision of the physician to start antibiotics. The study, therefore, is the ultimate combination of early-goal-directed therapy and reduction of antibiotic duration. Patients can be included within 24 hours after receiving their first dose of antibiotics. Informed consent has to be obtained in writing from the patient or his or her legal representatives prior to inclusion.

Exclusion criteria are: (1) the inability to acquire a written informed consent, or (2) when prolonged antibiotic therapy is indicated (>3 weeks, e.g. endocarditis, cerebral/hepatic abscess), (3) patients with severe infections due to viruses, parasites or tuberculosis, (4) patients entering the ICU for merely short-term post-operative observation, (5) patients with an estimated length of stay less than 24 hrs, (6) patients suffering from cystic fibrosis, (7) severely immunocompromised patients such as patients with HIV and a CD4 count of less than 200 cells/mm, neutropenic patients (<500 neutrophils/mL), (8) patients with solid organ transplantation, or (9) moribund patients.

The use of corticosteroids does not interfere with PCT measurements, while effects on CRP levels have been demonstrated [20]. Since the purpose of this study is to demonstrate the efficacy and safety of PCT-guided therapy in a real-life setting patients on corticosteroid therapy will not be excluded from the study. Use of systemic corticosteroids will be monitored and recorded throughout the trial.

### Treatment and intervention

The decision to start antibiotic treatment will in no way be affected by the trial. In all patients antibiotics will be started based on a clinical suspicion of infection or microbiological evidence of infection. This decision is fully at the discretion of the treating intensivist. Once antibiotics are administered for newly suspected or proven bacterial infection, patients or their legal representatives will be asked for informed consent. If informed consent is obtained, the patient will be randomised to either the standard therapy arm (control group) or the PCT arm (intervention group). Randomisation will be stratified for diagnostic group and study centre. When a patient is randomised for the control group, no PCT measurements will be performed. Other laboratory tests for infection such as CRP are allowed. When a patient is randomised for the PCT group, PCT will be measured at base-line called the T0-serum sample (T0: as close to initiation of antibiotics as possible, at least within 24 hrs). On the following days the ICU team will be provided with daily PCT values until ICU-discharge or until the third day after all systemic antibiotics have been discontinued. Along with daily PCT values a non-binding advice will be generated to consider stopping the prescribed antibiotics if PCT has decreased to <20% of its peak value (relative stopping threshold) or has reached a value of below 0.5 ng/ml (absolute stopping threshold). PCT levels will not be used to initiate antibiotic therapy. In case of two consecutive PCT levels below 0.5 ng/ml a stopping advice will be generated.

As for the control group, the physician will receive routine daily laboratory values as requested and no additional advice. Thus, treatment for both patient groups will be based on existing experience, expertise and local protocols, however, in the intervention group doctors are additionally provided with daily PCT levels, and a non-binding advice on continuation or discontinuation of antibiotic therapy by email, electronic patient data management system feedback, or research nurses. In both groups daily multidisciplinary reviewing will be performed on antibiotic therapy and duration, based on the clinical course, microbiological results and treatment guidelines. If doctors do not adhere to the stopping rules, reasons for non-adherence will be recorded.

### Data collection and management

Data management will be performed by the investigators or research nurses of the participating ICU. The investigators must ensure that the patient’s anonymity is maintained. The subjects will be identified by a trial identification number. The list containing the subjects name and allocation numbers are kept in strict confidence by the principal investigator. The database used for electronic data transfer of any clinical research file (CRF) or subject related data will be protected by a password. Data base integrity and data safety as well as privacy are warranted by the contracted research organisation, the Julius Center for Human Research, Utrecht, the Netherlands.

### Procalcitonin assays

PCT will be measured with various validated assays: the automated Kryptor platform (Thermo Fisher Scientific, Hennigsdorf, Germany), the Roche Elecsys Thermo Fisher Scientific PCT assay, the Siemens Centaur Thermo Fisher Scientific PCT assay or using BioMerieux’s Vidas Thermo Fisher Scientific PCT assay. The Thermo Fisher Scientific Kryptor sensitive PCT will be applied on this platform using Time Resolved Amplified Cryptate Emission (TRACE) technology and is based on a polyclonal antibody against calcitonin and a monoclonal antibody against katacalcin, which binds to the calcitonin and katacalcin sequence of the calcitonin pro-hormone. The test is considered a homogeneous immunoassay (sandwich principle) and is validated on serum and plasma (EDTA and heparin) matrix. The direct measuring range of the assay is from 0.02-50 ng/ml, with automated dilution extending the upper range to 1000 ng/ml. The Functional Assay Sensitivity (FAS) is 0.06 ng/ml.

The Roche Elecsys Thermo Fisher Scientific PCT assay also uses an immunoassay based on a sandwich principle based on a polyclonal antibody against calcitonin and a monoclonal antibody against katacalcin, which binds to the calcitonin and katacalcin sequence of the calcitonin prohormone. The direct measuring range of the assay is from 0.02-100 ng/ml. The Functional Assay Sensitivity (FAS) is 0.06 ng/ml. Procedure time of the assay is 18 minutes. The assay is validated on serum and plasma (EDTA and heparin) matrix.

The BioMerieux Vidas Thermo Fisher Scientific PCT assay is an Enzyme-Linked Fluorescent Assay (ELFA) involving a one-step immunoassay sandwich method using a Solid Phase Receptacle (SPR). The system uses a ready to use test strip into which a sample volume of 200 micro litres of serum or lithium heparin plasma is pipetted. The direct measuring range is 0.05-200 ng/ml. The FAS is determined to be 0.09 ng/ml.

Procedure time for all these assays is less than 30 minutes. Each participating centre will have access to a Kryptor machine, a suitable Vidas or Roche immunoanalyser to expedite the determinations and its adjunctive advice.

Whenever results exceed the maximum measuring range of any of the above described machines, manual dilution will be used, in accordance with the manufacturer’s specifications to achieve the true quantified value of a patient result. All patient results will be given in two decimals for the entire measuring range.

### Sample size and statistical analysis

Antibiotic duration is the main primary outcome of this on-going study. The expected standard deviation (SD) of antibiotic treatment, based on previous intervention trials [15-20] will be 6 days in both groups. Assuming a mean baseline antibiotic duration of 8 days, with a significance α-level of 5% and a power of 90%, 757 patients are needed in both groups. Patients that are discharged from the ICU before a stopping advice could have been issued and who are still being treated with antibiotics are considered “dropped out”. Assuming a drop-out rate of 20%, we need to include 908 patients in each group, so for the superiority margin in total 1816 patients will need to be included.

As the PCT intervention arm should be non-inferior, in terms of safety (mortality), in comparison to the standard treatment, the non-inferiority margin for PCT guided antibiotic management regarding 28-day-mortality is set on 8%. Based on previous trials, like the PRORATA-study [17], we assumed a 28% mortality rate in each group. With a one-sided significance α-level of 2.5% and a power of 90%, 714 patients are needed in both groups. Again with a drop-out of 20%, 1714 patients will need to be included. With such a sample size (and alpha 5% and beta of 20%) we would have been able to detect a 15% relative increase in mortality. Such conventional boundaries are very acceptable in most trials investigating the efficacy of new drugs. Here, we aim to do better, with an alpha of 2.5% and beta of 10%.

Losses to follow-up on both arms will be registered including the reason for loss to follow-up. Furthermore, the primary endpoint will be explored for association with potential prognostic factors in a survival analysis. The factors that will be considered are age, sex, APACHE IV-score, SOFA-score and usage of corticosteroids or dialysis.

The primary analysis population will consist of all randomized patients following the intention-to-treat principle. For the primary analysis, losses to follow-up (in both arms) will be included in the 28-day analysis of mortality and antibiotic consumption. The primary analysis will be repeated with the subset of patients who complied with the stopping threshold, as so-called “per protocol analysis” of mortality and antibiotic consumption on day 28.

### Interim analysis

The first interim analysis was performed after enrolment of 750 patients. Preceding this analysis the non-inferiority margin was set at 8% and a p-value of 0.0294 was used, corresponding with the performance of one interim-analysis in accordance with the Pocock method [21]. The DSMB examined the data and the trial would have been stopped immediately if the above predefined margins were reached. Furthermore, the DSMB reviewed the trial’s progress and adverse events according to treatment assignment.

### Duration

With the current inclusion rate of 50 patients per month, the total duration time is estimated at 36 months and the last patient is expected to be included around May 2013

## Discussion

### Published studies

The additional prognostic value of PCT in some critically ill patients has repeatedly been demonstrated. Until now six randomized controlled trials have been published that addressed the effectiveness and safety of PCT-guided strategy in antibiotic treatment of septic patients in adult intensive care units (ICU’s) [15-19,22], Several systemic reviews have been performed [23-28] and even an individual patient data meta-analysis in acute respiratory infections was published recently [14]. Thus, the question arises whether another study is warranted. The six trials were done with either small sample sizes or within highly selected populations [15-20]. Hochreiter [16] and Schroeder [8] both included patients after abdominal surgery. Svoboda et al. investigated 72 patients after abdominal surgery or surgery for major multiple trauma, using a semi-quantitative PCT assay [19]. Stolz et al. included 101 patients with only ventilator-associated-pneumonia [18]. Schuetz and colleagues have performed a individual patient data meta-analysis in patients with an acute respiratory infection [14]. This study combined 4550 patients with a suspicion of respiratory infection of which 598 patients were admitted to the ICU. Even in these severely ill patients the median duration of antibiotic treatment was reduced from 12 days to 8 days (P < 0.001). The two remaining studies have been published with a heterogeneous adult ICU population [15,17]. Remarkably, Nobre et al. excluded 203 out of 282 patients [15]. Due to strict exclusion criteria, this trial included only 79 patients.

Therefore, the PRORATA-study appears to be the only study with a larger number of adult patients in a heterogeneous critically ill population [17]. This study was designed as a both a start and stop study and comprised predominantly non-surgical patients (10% were surgical). Within the study a clear reduction of 2.7 days in antibiotic treatment duration was seen in the PCT arm. However, concerns exist about mortality statistics, the lack of power, and potential treatment bias. Furthermore, it is important to recognise that this study was performed in France, with one of the highest baseline antibiotic consumption rates in Europe. Therefore, it is important to investigate, in particular in a country with a relatively low antibiotic consumption, if PCT-guidance will lead to reduction of antibiotic use.

More recently, the PASS study was published [22]. This study was not intended to reduce antibiotic duration. The objective was to analyse whether PCT guidance was able to detect an infectious deterioration and could lead to improvement of the 28-day survival in 1200 ICU patients [22]. This trial did not show that a PCT-guided algorithm lead to improved outcome. In contrast, patients in the PCT arm had a longer LOS and used more antibiotics than the patients in the control arm.

### SAPS-trial

The SAPS study was conceived to include a heterogeneous ICU patient population in a real life setting, focusing only on the additional value of PCT in guiding antibiotic treatment. The rather large sample-size (over 1800 patients) allows for adequate testing of both efficacy (consumption of antibiotics) and safety (mortality, recurrent infections). This is a multicenter study (16 centres) with an estimated high recruitment rate (5 patients/month in a 10-bed ICU). The anticipated antibiotics use in both trial arms will be low compared to the studies from France or the US. Patients on selective decontamination of the digestive tract (SDD) will not be excluded. Another important aspect of the SAPS trial will be costs-analysis. So far only one study gathered data on costs of the PCT-based strategies in the ICU; however this study enclosed only 27 patients, who all underwent abdominal surgery [19]. When antibiotics are reduced by PCT-guided therapy, economic aspects should certainly be considered, acknowledging that PCT is more expensive than common laboratory biomarkers like CRP. As has been repeatedly noted by authors critical of PCT as a sole indicator of sepsis, PCT should be viewed together with other information, including fever, leukocyte count and very importantly, CRP. Irrespective of the relative merits of CRP and PCT, the *de facto* situation is that most ICU’s regularly perform the relatively inexpensive CRP-assay. This is also the case in ICUs that participate in the SAPS-study. Thus, in the PCT-arm of the SAPS-study, clinicians will inevitably judge PCT and CRP levels in conjunction. It will thus be of great interest if the PCT-assay that is currently considerably more expensive than CRP, making up for its costs in reducing antibiotic costs.

SAPS therefore not only aims to address issues of effectiveness or safety, but also unanswered questions regarding cost-effectiveness.

It should be stressed that when antibiotics are reduced by a PCT-supported therapy, the safety of such an intervention is paramount. Only without an increase in mortality during a long-term follow-up for the PCT-guided group, can this intervention prove itself to be acceptable. In the aforementioned studies no statistically significant difference in mortality was found between patients managed with PCT-guided algorithms versus ‘standard of care’. However, in the PRORATA-study the non-inferiority margin was set on 10% (absolute increase), and within this margin no statistical significant effect on mortality was noted. However, the 60-day mortality was 3.8% higher in the PCT-guided group. With a 90% confidence interval, the lower limit was 9.7%, approaching the preset 10% margin, which led to some criticism [28]. The authors proclaimed, however, that this non-inferiority margin in a real life setting was in accordance with international recommendations for non-inferiority trials assessing antibiotic treatment for severe community-acquired pneumonia or for assessment of new antibacterials. However, an increase of mortality from 5-10% [29] to 20% in community-acquired pneumonia would not be considered acceptable. The SAPS-study will set the non-inferiority margin at 8%. Theoretically, an even lower margin will be favourable, however this would require an unrealistic sample size with several thousands of patients and thereby rendering such an investigation, unfortunately, unenforceable.

Another aspect is optimisation of PCT cut-off values that are applicable in the intensive care department. There is still an on-going discussion about what cut-offs should be used for PCT to optimise antibiotic therapy. In SAPS we apply stopping rules similar to those used in the PRORATA-trial. Although some may considerer the PRORATA and SAPS stopping rules conservative, we consider it prudent to carefully examine the clinical value of this rule through trials, before considering new rules. Finally, even if the primary endpoints of this trial are not achieved, this trial will still be of great value, as it will provide important insights in the optimisation of antibiotic therapy in Dutch ICUs.

## Competing interests

This is an investigator-initiated and investigator-driven study. No commercial sponsor had or will have any involvement in design and conduct of the studies (i.e., main project and ancillary projects of the SAPS study), namely collection, management, analysis, and interpretation of the data; and preparation, decision to submit, review, or approval of the manuscript. AB, DdL and JvO all received speaking fees from Brahms GmbH. All other contributors and authors have not disclosed any conflicts of interests.

## Authors’ contributions

EAdJ, DdL, JvO, MN and AB designed, wrote the protocol and initiated the study. EdJ, DdL, JvO, MN and AB managed the trial, included patients and collected data. JT co-wrote the statistical paragraph. All authors amended and approved on the final manuscript.

## Authors’ information

The steering committee

The SAPS steering committee consists of the following:

Albertus Beishuizen (chair), Dylan de Lange, Jos van Oers, Evelien Assink-de Jong, Maarten Nijsten

Data Safety & Monitoring Board:

W. M. van den Bergh (Utrecht) J. M. Daniels (Amsterdam), M. Heijmans (Amsterdam)

Participating sites:

VU University medical center, Amsterdam [A. Beishuizen, E. Assink-de Jong]

University Medical Center Utrecht, Utrecht [D. de Lange]

St. Elisabeth Hospital, Tilburg [J. van Oers]

St. Lucas Andreas Hospital Amsterdam [M. van den Elsen]

Isala Clinics, Zwolle [H. Kieft]

Diakonessenhuis, Utrecht [H. Endeman, L. van Lelyveld]

Martini Hospital, Groningen [B. Loef]

Atrium Medical Center, Heerlen [T. Dormans]

Medical Center Haaglanden, Den Haag [G. Admiraal]

Bronovo Hospital, Den Haag [J. Streefkerk]

WestFriesGasthuis, Hoorn [A. Tacx]

Slotervaart Ziekenhuis, Amsterdam (G. Kluge, H. Biermann]

University Medical Center Groningen, Groningen [M.N. Nijsten]

Medisch Spectrum Twente, Enschede [A. Beishuizen]

Wilhelmina Ziekenhuis, Assen [J. Lutisan]

Canisius-Wilhelmina Ziekenhuis, Nijmegen [J. Schouten]

## Pre-publication history

The pre-publication history for this paper can be accessed here:

http://www.biomedcentral.com/1471-2334/13/178/prepub
